# Serum metabolic profiling analysis of Gitelman syndrome using untargeted metabolomics

**DOI:** 10.1080/0886022X.2026.2662094

**Published:** 2026-04-29

**Authors:** Jian Han, Lanxin Ma, Heng Luo

**Affiliations:** Precision Medicine Center, People’s Hospital of Chuxiong Yi Autonomous Prefecture, Chuxiong, China

**Keywords:** Gitelman syndrome, metabolomics, lipid metabolism, *SLC12A3*

## Abstract

Gitelman syndrome (GS) is a rare hereditary kidney disorder characterized by electrolyte abnormalities. However, its systemic manifestations, such as growth retardation, suggest broader metabolic disturbances. To investigate this issue, we explored the link between GS and lipid metabolism, hypothesizing that the genetic mutation affects pathways beyond renal function. Our study focused on five pediatric GS patients carrying homozygous *SLC12A3* c.1262G > T mutation and seven healthy, age-matched controls from the Yi ethnic group. Consistent with systemic involvement, these children exhibited impaired growth and significantly lower serum total cholesterol compared to healthy controls. To determine whether this mutation directly causes cholesterol metabolism abnormalities, we conducted *in vitro*. 293 T cell with the c.1262G > T point mutation showed reduced intracellular cholesterol storage and increased cholesterol efflux, confirming a direct role of the mutation in disrupting cholesterol metabolism. Returning to our patient samples, we performed untargeted serum metabolomics to obtain a comprehensive view of metabolic changes. This analysis revealed alterations in numerous lipid-related metabolites. Crucially, glycerophospholipid metabolism was identified as the most significantly perturbed pathway in GS patients. These findings provide the first clear evidence linking this renal tubulopathy to systemic dyslipidemia, fundamentally expanding our understanding of its pathophysiology.

## Introduction

Gitelman syndrome (GS; Online Mendelian Inheritance in Man [OMIM] #263800) is a rare autosomal recessive renal tubular disorder primarily caused by mutations in the *SLC12A3* gene. These mutations result in partial or complete loss of function of the encoded thiazide-sensitive sodium-chloride cotransporter (NCC), impairing Na^+^ and Cl^-^ reabsorption in the distal convoluted tubule. Consequently, affected individuals present with hypokalemia, hypomagnesemia, hypocalciuria, and metabolic alkalosis [1]. The prevalence of GS in European populations is estimated to range from 1 to 10 per 40,000 individuals. Although data on Asian populations are limited, some studies suggest that the prevalence may be higher [2,3]. Traditionally, the diagnosis of GS relies on the evaluation of serum and urinary electrolyte levels, along with confirmatory genetic testing [4].

Although GS is primarily characterized by electrolyte abnormalities, we observed that several pediatric patients with GS exhibited growth retardation and a lower Body Mass Index (BMI) compared to their age-matched peers. Such systemic clinical signs suggest that the consequences of *SLC12A3* mutations extend beyond renal tubule function to impact global energy metabolism. Although metabolites are generally stable within biological systems, their concentrations can vary markedly in response to external stimuli. Such fluctuations reflect underlying regulatory and pathophysiological processes, thereby providing valuable insights into disease mechanisms [5]. We utilized untargeted metabolomics, a powerful approach for capturing systemic biochemical changes, to explore potential alterations in metabolic pathways, particularly those related to lipid metabolism, in pediatric patients with GS.

## Materials and methods

### Patient recruitment

Participants were recruited from four families affected by GS within the Yi ethnic group in Yunnan, China. All affected individuals were receiving care at the People’s Hospital of Chuxiong Yi Autonomous Prefecture. In parallel, seven healthy, age-matched children from the same geographic area were enrolled as wild-type (WT) controls. The study was conducted in accordance with human research guidelines stated in the Declaration of Helsinki and approved by the Ethics Committee of Chuxiong Yi Autonomous Prefecture People’s Hospital (KYCS2024017). Additionally, the study complied with the regulations issued by the Ministry of Science and Technology of the People′s Republic of China regarding the review and approval of human genetic resources.

### Gene mutation identification

Whole-exome sequencing (WES) was performed on all patients using the MGISEQ-2000 platform (MGI Tech) and validated by Sanger sequencing. Variants were filtered and annotated using the following databases: 1000 Genomes Project (http://www.1000genomes.org), Genome Aggregation Database (gnomAD) (http://gnomad-old.broadinstitute.org), ClinVar (https://www.ncbi.nlm.nih.gov/clinvar/), Online Mendelian Inheritance in Man (OMIM) (http://omim.org/), and the Human Gene Mutation Database (HGMD) (http://www.biobase-international.com/product/hgmd). The pathogenicity of candidate variants was assessed per the guidelines of the American College of Medical Genetics and Genomics (ACMG) [6].

The p.C421F mutation in the *SLC12A3* gene (ENST00000438926, NM_000339.3) was validated by Sanger sequencing. The primer sequences used were as follows: primer-F: GTACTTATCATGGCTCCGGG; primer-R: GACAGACCTGGGGCTCTGAAC.

DNA amplification was performed following this protocol: an initial denaturation at 95 °C for 10 min, followed by 35 cycles of denaturation at 95 °C for 30 s, annealing at 60 °C for 30 s, and extension at 72 °C for 60 s. A final extension was performed at 72 °C for 10 min. Sanger sequencing was performed using a 3500xL Dx Genetic Analyzer (Applied Biosystems).

### Fasting lipid profile assays

Serum total cholesterol (TC) levels in patients with GS and normal subjects were measured using a Siemens ADVIA2400 automatic biochemical analyzer.

### Cell line and culture condition

The 293 T cell line was obtained from the American Type Culture Collection (ATCC). Cells were maintained in high-glucose Dulbecco’s Modified Eagle’s Medium (HyClone) supplemented with 10% fetal bovine serum (Gibco) and penicillin-streptomycin (Gibco). The cultures were incubated at 37 °C in a humidified atmosphere containing 5% CO_2_.

The 293 T cell line with the *SLC12A3* c.1262G > T (p.C421F) point mutation was custom-generated by UBIGENE (Contract ID: UBISUM250617ZJ1). The stable cell line was established *via* electroporation (1200 V, 30 ms, 2 pulses) using a gRNA targeting SLC12A3 (5′-GAAGTTCCAGCCATAGCTGCAGG-3′). PCR and Sanger sequencing verified the successful isolation of the homozygous (HOM) clone and a corresponding WT control clone. Prior to subsequent analyses, both cell lines were cultured for 48 h.

### Preparation of cell lysates and supernatants

Following transfection, the culture medium was aspirated, and the 293 T cells were washed once with ice-cold PBS (Beyotime, C0221A). To prepare cell lysates, BeyoLysis^™^ Buffer A for Metabolic Assay (Beyotime, S0211S) was added at a ratio of 100–200 µL per 2 × 10^5^ to 1 × 10^6^ cells. The cells were then detached by gentle pipetting and transferred to a microcentrifuge tube. After a 15-min incubation on ice, the mixture was centrifuged at 12,000 × g for 3–5 min at 4 °C. The resulting supernatant and cell lysate were carefully collected for subsequent analysis. For the collection of cell culture supernatants, the culture medium was aspirated directly and centrifuged under the same conditions to pellet any cellular debris. The cleared supernatant was then collected for analysis.

### Quantification of cholesterol and cholesteryl esters

The concentrations of total cholesterol, free cholesterol, and cholesteryl esters were quantified using the Cholesterol and Cholesteryl Ester Assay Kit (Beyotime, S0211S) according to the manufacturer’s instructions. Briefly, for the total cholesterol assay, diluted cell lysates and supernatants were mixed with the provided total cholesterol working solution and incubated for 30 min at 37 °C, protected from light. For the free cholesterol assay, the diluted samples were similarly mixed with the free cholesterol working Solution and incubated under the same conditions.

The concentration of cholesteryl esters was determined by subtracting the free cholesterol concentration from the total cholesterol concentration. The absorbance for all assays was measured at 570 nm, and the final concentrations were calculated based on a cholesterol standard curve.

## Untargeted metabolomics

A total of 100 μL of serum from 5 GS HOM patients and 7 WT was mixed with 300 μL of methanol, vortexed for 1 min, and then centrifuged at 12,000 rpm for 10 min at 4 °C. Next, 300 μL of supernatant was transferred to a new tube and dried under vacuum at low temperature. After drying, 100 μL of 50% methanol was added, and the mixture was reconstituted by sonication. Following this, 90 μL of supernatant was placed in a liquid-phase vial for measurement. The same volume of supernatant was obtained by centrifugation at 12,000 rpm for 10 min at 4 °C and transferred into a liquid-phase vial for analysis. Additionally, equal volumes from each sample were mixed homogeneously to prepare quality control (QC) samples.

Sample metabolites were separated using an ACQUITY UPLC HSS T3 reversed-phase column (1.8 μm, 2.1 mm × 100 mm; Waters Corporation) on a Dionex UltiMate 3000 ultra-fast liquid chromatograph (Thermo Scientific). The chromatographic conditions were as follows: mobile phase A consisted of water with 0.1% formic acid, and mobile phase B was methanol. The flow rate was set to 0.3 mL/min, with an injection volume of 4.0 μL and a column temperature of 50 °C. The elution gradient is detailed in Table 1.

**Table 1.** Reversed-phase chromatographic elution gradient.

Time (min)	A (v%)	B (v%)
0	98.0	2.0
1.0	98.0	2.0
5.5	0	100
14	0	100
14.1	98.0	2.0
16.0	98.0	2.0

Further analyses were performed using a Q Exactive quadrupole orbiting ion trap high resolution mass spectrometer (Thermo Scientific) equipped with a heated electrospray ionization (HESI) source. The mass spectrometry parameters were set as follows: spray voltage of 3.7 kV for positive ion mode and 3.5 kV for negative ion mode; capillary temperature set to 320 °C; sheath gas pressure at 30 psi; auxiliary gas pressure at 10 psi; vaporizer temperature at 300 °C; and both sheath and auxiliary gas were nitrogen. The collision gas was nitrogen at 1.5 mTorr. Full MS scans were acquired using the following parameters: resolution of 70,000; automatic gain control (AGC) target of 1 × 10^6^; and maximum injection time of 50 ms. External mass calibration was performed, resulting in a mass accuracy of 5 ppm. Data-dependent MS/MS (dd-MS^2^) scans were performed using these parameters: resolution of 17,500; AGC target of 1 × 10^5^;s maximum injection time of 50 ms; TopN of 10 (dynamic exclusion); isolation window of 2 m/z; and normalized collision energies of 10, 30, and 60 V.

### Data processing

Raw data were imported into Progenesis QI software for peak alignment, peak picking, and deconvolution, generating a data matrix that included retention time, mass-to-charge ratio (m/z), and peak intensity. Various addition ions (e.g., [*M* +* H*]^+^ and [*M*+*Na*]^+^) were deconvoluted to individual ion features. Furthermore, ion features with a coefficient of variation (CV) > 15% in the QC samples were excluded to ensure the reliability and reproducibility of metabolite detection. Metabolite identification was performed by matching the accurate mass (monoisotopic mass) to entries in the Human Metabolome Database (HMDB, https://hmdb.ca/) and the LIPID MAPS Structure Database (LMSD, https://www.lipidmaps.org/).

### Analysis of the Receiver Operating Characteristic Curve (ROC)

Receiver operating characteristic (ROC) curves are commonly used to assess the performance of binary classification models. Sensitivity (true positive rate) was plotted on the y-axis, while 1 - specificity (false positive rate) was plotted on the x-axis. The area under the curve (AUC) was used to evaluate the metabolite’s ability to differentiate between groups. A higher AUC value indicates a greater discriminatory power of the metabolite in differentiating between WT and HOM groups.

### Statistical analysis

Data were analyzed using GraphPad Prism 9.0. Unpaired t-tests were performed, and *p <* 0.05 was considered statistically significant. Data and error bars represent the mean ± SD. The Benjamini-Hochberg procedure was used to control the FDR. Differences in age between the patient group and the healthy control group were assessed using Pearson correlation analysis. A *p*-value of less than 0.05 was considered statistically significant.

## Results

### Phenotype of the patients

**Figure 1.**
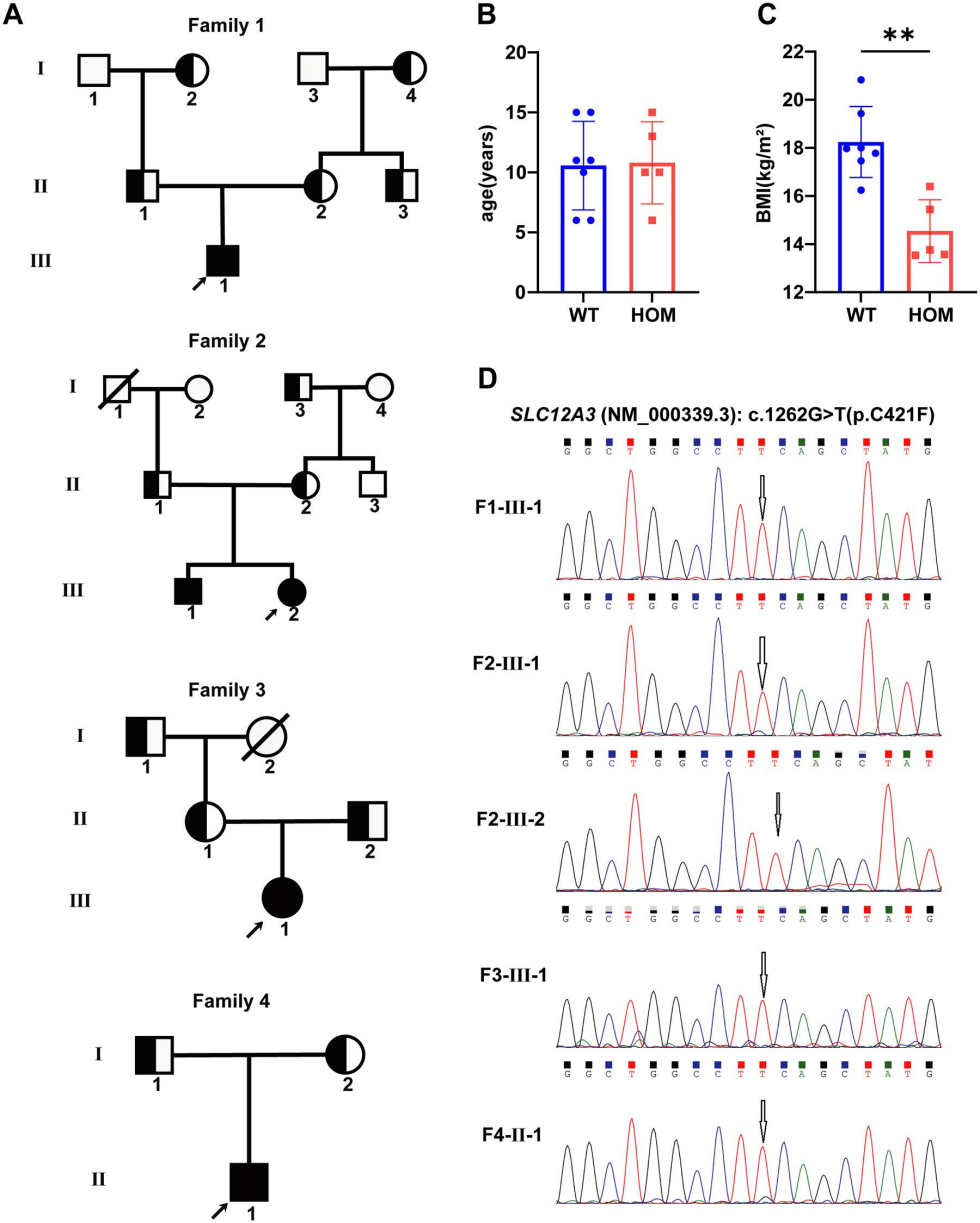
Pedigree and mutation analysis of the family. (A) Pedigrees of the GS families. The square represents male, and the circle represents female. The arrow represents the proband. The shaded symbol represents a homozygous (HOM) patient, the unshaded symbol represents wild-type (WT) individuals, and the half-shaded symbol represents a heterozygous patient. Draw a diagonal line through the square or circle represents the member is deceased. Age (B) and Body Mass Index (BMI) (C) of GS homozygous patients (*n* = 5) and wild type individuals (*n* = 7), **p < 0.01. (D) Sanger sequencing validation of the *SLC12A3* mutation (c.1262G > T) in the GS patients.

All five patients with GS carried a homozygous *SLC12A3* (NM_000339.3): c.1262G > T(p.C421F) mutation, which was also verified by Sanger sequencing (Figure 1(A and D)).

Patient 1 (F1-III-1) was first hospitalized at age four due to intestinal dysfunction and polydipsia as the initial presenting symptom. Hypokalemia was later identified and treated symptomatically. The patient subsequently experienced recurrent episodes of weakness and hypokalemia, with the lowest recorded potassium level being 2.19 mmol/L. At 6.3 years of age, genetic testing confirmed a diagnosis of GS.

Patient 2 (F2-III-1) developed symptoms of limb weakness at 12 years of age, primarily manifesting as an inability to stand or hold objects, with a minimum blood potassium level of 1.93 mmol/L. He was treated symptomatically for ‘hypokalemia’ at other medical institution. At 16 years of age, the patient was admitted to our hospital for comprehensive management. Physical examination revealed that the patient’s height was more than three standard deviations below the mean height for his age and sex. Renal ultrasound revealed small kidney stones. The estimated glomerular filtration rate (eGFR) was 92.97 mL/min/1.73m^2^. A diagnosis of GS was established during the current study.

Patient 3 (F2-III-2) had been experiencing ‘recurrent fatigue’ for over 10 years. These episodes were often accompanied by hypokalemia, for which the patient received potassium supplementation multiple times, resulting in occasional improvement. Genetic testing conducted during the current study confirmed a diagnosis of GS.

Patient 4 (F3-III-1) began experiencing recurrent episodes of limb weakness at the age of 15. The symptoms were initially characterized by finger spasms and lower extremity weakness, which, in severe cases, progressed to an inability to stand or walk. Genetic testing conducted in this study confirmed the diagnosis of GS.

Patient 5 (F4-II-1) first presented at the age of 12 with symptoms including weakness in the extremities, predominantly in the lower limbs. The patient was initially treated at a local clinic. Genetic testing conducted in this study confirmed the diagnosis of GS.

Detailed information on the clinical presentation and *SLC12A3* gene mutations for the five patients with GS is provided in Table 2.

**Table 2.** Clinical presentation and *SLC12A3* gene mutations of GS patients.

Individual	F1-III-1	F2-III-1	F2-III-2	F3-III-1	F4-II-1
Gender	Male	Male	Female	Male	Male
Age (years)	6	10	10	13	15
Height/weight (m/kg)	1.07/15.5	1.3/26.1	1.25/21.5	1.33/29	1.66/37.4
Blood pressure	78/54	100/71	104/62	95/56	101/70
Potassium (3.6–5.0 mmol/L)	2.51	1.93	2.11	2.33	2.15
Chloride (98–107mmol/L)	102.30	101.00	99.80	99.10	96.30
Magnesium (0.70–1.00 mmol/L)	0.54	0.66	0.52	0.51	0.49
Aldosterone	High	Normal	High	Normal	Normal
Renin	High	High	High	Normal	High
eGFR^a^	82.52	92.97	92.97	96.51	91.33
Fasting blood glucose (3.9–6.1 mmol/L)	4.62	5.50	4.70	5.70	4.00
Nucleotide change	c.1262G > T	c.1262G > T	c.1262G > T	c.1262G > T	c.1262G > T
Mutation type	HOM	HOM	HOM	HOM	HOM

^a^eGFR: Calculated using the Cockcroft-Gault formula.

There was no statistically significant difference in age between children in the HOM and WT groups (*p* > 0.05) (Figure 1(B)). The mean BMI was 15.02 kg/m^2^ for the HOM group and 18.37 kg/m^2^ for the healthy control group. The BMI of the WT group was significantly higher than that of the HOM group (*p* < 0.05) (Figure 1(C)).

### Non-targeted metabolome quality control assessment

**Figure 2.**
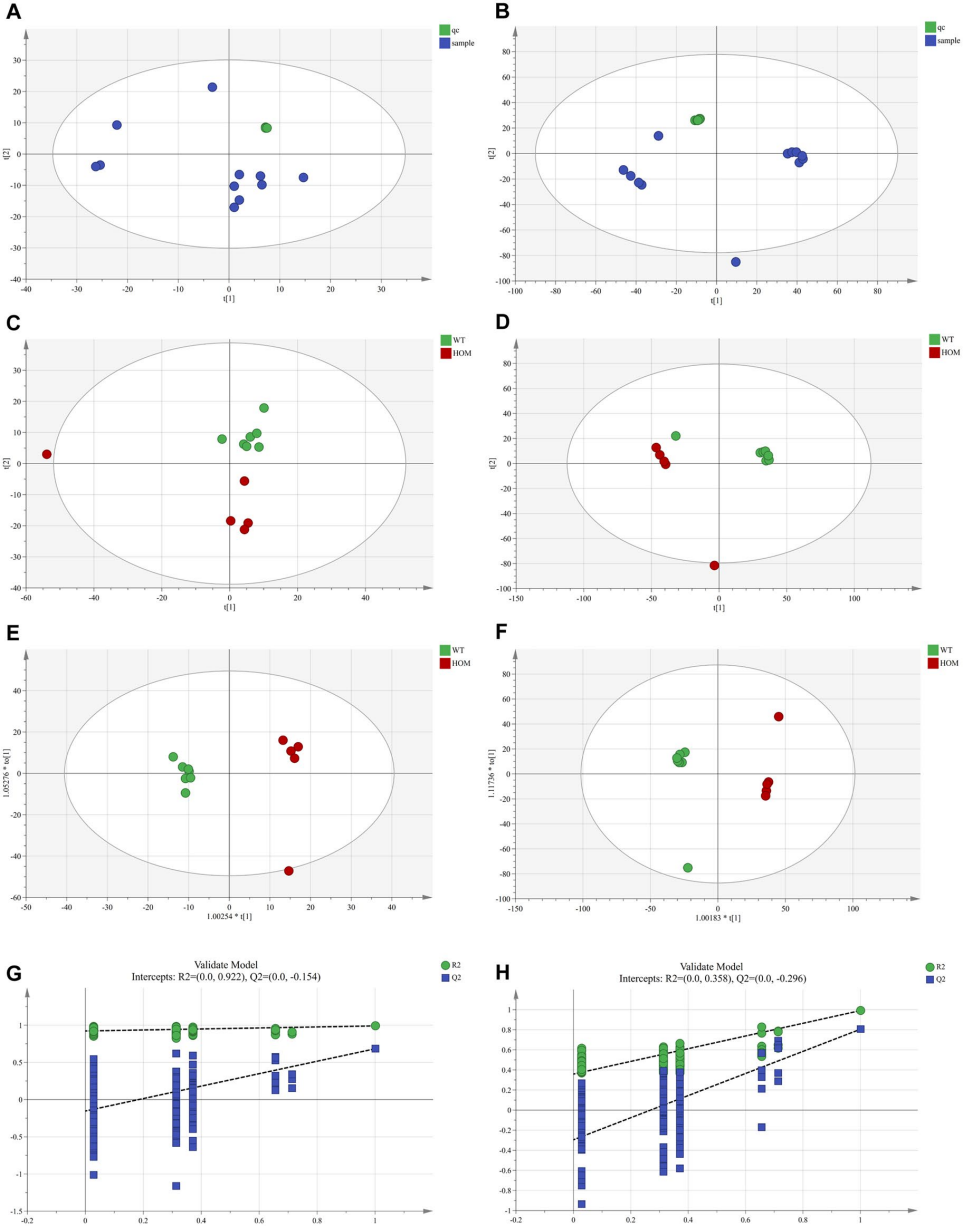
Gitelman syndrome model evaluation. (A) Plot of PCA scores for negative ion modes (with QC). (B) Plot of PCA scores in positive ion mode (with QC). (C) Plot of PCA scores for negative ion mode. (D) Plot of PCA scores in positive ion mode. (E) Plot of OPLS-DA scores for negative ion mode. (F) Plot of OPLS-DA scores for the positive ion model. (G) Negative ion model OPLS-DA model replacement test plot. (H) Positive ion mode OPLS-DA model substitution test plot.

Principal component analysis (PCA), an unsupervised technique, was used to evaluate sample quality. QC data obtained from multiple injections of the same sample were included in this assessment. In the PCA score plots for both positive and negative ion modes (Figure 2(A–B)), the QC samples clustered tightly, with most points falling within the 95% confidence interval, indicating good system reproducibility. Therefore, the acquired data were deemed suitable for further analysis.

### Non-targeted metabolome sample methodological assessment

To assess the metabolic differences within and between sample groups, the data were normalized to enhance interpretability and reliability. The normalized data were then analyzed using Simca 14.1 software. PCA was applied to assess the model’s explanatory power (R^2^X) and predictive accuracy (Q^2^). For the normalized dataset, the PCA model in positive ion mode yielded an R^2^X of 0.871 and a Q^2^ of 0.665, while in negative ion mode, the R^2^X was 0.537 and the Q^2^ was 0.01. These results suggest good model reproducibility (Figure 2(C–D)).

Orthogonal partial least squares discriminant analysis (OPLS-DA) was employed to validate the model and identify significantly different metabolites. An R^2^Y value close to 1 indicates a high degree of explained variance between the groups, and thus, a greater difference between them. For the positive ion mode, the OPLS-DA model yielded an R^2^Y of 0.993 and a Q^2^ of 0.875. For the negative ion mode, the R^2^Y was 0.987, and the Q^2^ was 0.565. The OPLS-DA score plots demonstrated strong explanatory power and acceptable predictability, indicating that the model accurately described the acquired data. Therefore, the model was considered stable and valid for subsequent screening of differential metabolites (Figure 2(E–F)).

To prevent overfitting in the supervised model, an external validation was performed using the OPLS-DA model permutation test. The intercepts of the regression lines on the vertical axis for the Q^2^ values in both the positive and negative ion modes were less than 0, indicating that the model was not overfitted (Figure 2(G–H)).

### Differential metabolite screening

**Figure 3.**
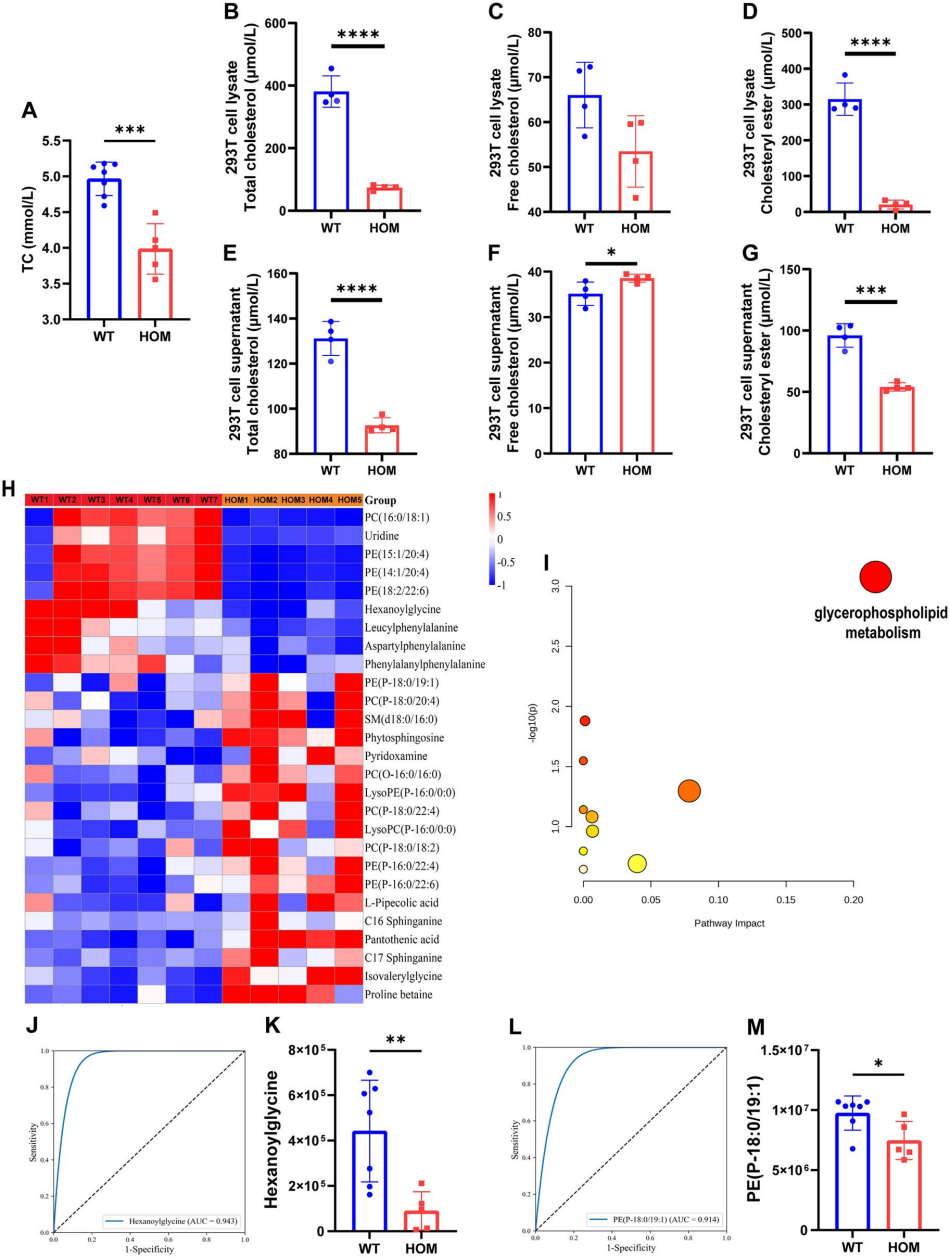
Metabolomic analysis reveals aberrant lipid metabolism in homozygous GS patients. (A) Comparison of serum total cholesterol (TC) levels between the WT and HOM groups. (B-D) Quantification of total cholesterol (B), free cholesterol (C), and cholesteryl ester (D) levels in lysates of 293 T cells expressing WT or HOM mutant *SLC12A3*. (E-G) Quantification of total cholesterol (E), free cholesterol (F), and cholesteryl ester (G) levels in supernatant of 293 T cells expressing WT or HOM mutant *SLC12A3*. (H) Heatmap illustrating the differential metabolites in serum samples from the WT and HOM GS groups. Each column represents an independent biological replicate, and each row corresponds to a specific metabolite. Red and blue indicate upregulated and downregulated relative abundances of the metabolites, respectively. (I) KEGG pathway enrichment analysis was performed on the differential metabolites. The analysis revealed that glycerophospholipid metabolism was the most significantly affected pathway. In the bubble plot, the significance of pathway enrichment is indicated by the y-axis position and color scale, while the pathway impact factor is represented by the x-axis position and the size of the bubble. (J, K) ROC curve (J) and statistical analysis (K) for hexanoylglycine. (L, M) ROC curve (L) and statistical analysis (M) for PE(P-18:0/19:1). * *p <* 0.05, ** *p <* 0.01, *** *p <* 0.001, **** *p <* 0.0001.

Serum TC levels were measured and compared between the GS and the healthy control group. The results revealed a significant reduction in serum TC levels in the GS group compared to controls (*p <* 0.05) (Figure 3(A)).

To investigate the impact of the *SLC12A3* (NM_000339.3): c.1262G > T (p.C421F) mutation on cellular lipid metabolism, we measured the levels of total cholesterol, free cholesterol, and cholesteryl esters in both the lysates and supernatants of 293 T cells transfected with WT or HOM mutant *SLC12A3*.

First, we examined the lipid profile in the 293 T cell lysates. The results revealed that, compared to the WT group, the HOM group exhibited significantly lower levels of total cholesterol in cell lysates (Figure 3(B)). Regarding free cholesterol, a decreasing trend was observed in the HOM group lysates, although the difference was not statistically significant (Figure 3(C)). Notably, the levels of cholesteryl esters were also significantly lower in the HOM group lysates than in the WT group (Figure 3(D)).

Subsequently, we analyzed the lipid profile of the cell supernatants. Similar to the lysates, total cholesterol levels were significantly lower in the supernatants from the HOM group compared to the WT group (Figure 3(E)). Interestingly, the level of free cholesterol was significantly higher in the supernatants of the HOM group than in the WT group (Figure 3(F)). Meanwhile, the levels of cholesteryl esters were also significantly lower in the HOM group supernatants (Figure 3(G)).

Differential metabolites in the OPLS-DA model were identified based on the following criteria: (1) variable importance in the projection (VIP) score: VIP > 1; and (2) *p*-value from a *t*-test: *p <* 0.05. Based on these criteria, 27 differential metabolites were identified between the GS and WT groups (Figure 3(H)). These metabolites were selected following multiple testing correction. Detailed information on the identified metabolites and the screening criteria is presented in Table 3. Among the identified metabolites, notable enrichments were observed for hexanoylglycine (HMDB0000701), PC (16:0/18:1) (LMGP01010005), PE (P-16:0/22:6) (LMGP02030001), LysoPE (P-16:0/0:0) (LMGP02070001), LysoPC (P-16:0/0:0) (LMGP01070006), and pantothenic acid (HMDB0000210).

**Table 3.** Differences in serum metabolites between the GS and normal control groups.

Compound	Formula	VIP	*p*-value	FDR	Accepted compound ID
PC(16:0/18:1)	C_42_H_82_NO_8_P	1.34589	0.02299	0.038392	HMDB0007972
Uridine	C_9_H_12_N_2_O_6_	1.16499	0.02299	0.038392	HMDB0000296
PE(15:1/20:4)	C_40_H_70_NO_8_P	1.31457	0.005766	0.013827	LMGP02010497
PE(14:1/20:4)	C_39_H_68_NO_8_P	1.27343	0.005766	0.013827	LMGP02010444
PE(18:2/22:6)	C_45_H_74_NO_8_P	1.21937	0.005766	0.013827	LMGP02011191
Hexanoylglycine	C_8_H_15_NO_3_	1.08979	0.004643	0.013973	HMDB0000701
Leucylphenylalanine	C_15_H_22_N_2_O_3_	1.099507	0.005766	0.013827	HMDB0013243
Aspartylphenylalanine	C_13_H_16_N_2_O_5_	2.05808	0.02299	0.038392	HMDB0000706
Phenylalanylphenylalanine	C_18_H_20_N_2_O_3_	1.090718	0.023459	0.039033	HMDB0013302
PE(P-18:0/19:1)	C_42_H_82_NO_7_P	1.035217	0.042217	0.046741	LMGP02030051
PC(P-18:0/20:4)	C_46_H_84_NO_7_P	1.035311	0.044942	0.049646	HMDB0011253
SM(d18:0/16:0)	C_39_H_81_N_2_O_6_P	1.035794	0.042436	0.047408	HMDB0010168
Phytosphingosine	C_18_H_39_NO_3_	1.073429	0.009366	0.020656	HMDB0004610
Pyridoxamine	C_8_H_12_N_2_O_2_	1.041167	0.035019	0.042213	HMDB0001431
PC(O-16:0/16:0)	C_40_H_82_NO_7_P	1.046772	0.021113	0.037721	LMGP01020029
LysoPE(P-16:0/0:0)	C_21_H_44_NO_6_P	1.049522	0.001556	0.005705	HMDB0011152
PC(P-18:0/22:4)	C_48_H_88_NO_7_P	1.046278	0.025026	0.039161	HMDB0011259
LysoPC(P-16:0/0:0)	C_24_H_50_NO_6_P	1.04233	0.034756	0.041753	HMDB0010407
PC(P-18:0/18:2)	C_44_H_84_NO_7_P	1.049453	0.039542	0.044405	HMDB0011244
PE(P-16:0/22:4)	C_43_H_78_NO_7_P	1.053184	0.014851	0.035919	LMGP02030033
PE(P-16:0/22:6)	C_43_H_74_NO_7_P	1.054053	0.039195	0.044117	LMGP02030001
L-Pipecolic acid	C_6_H_11_NO_2_	1.057147	0.034756	0.041753	HMDB0000716
C16 Sphinganine	C_16_H_35_NO_2_	1.051782	0.02299	0.038392	LMSP01040001
Pantothenic acid	C_9_H_17_NO_5_	1.5431	0.005766	0.013827	HMDB0000210
C17 Sphinganine	C_17_H_37_NO_2_	1.073169	0.005766	0.013827	LMSP01040003
Isovalerylglycine	C_7_H_13_NO_3_	1.00189	0.005766	0.013827	HMDB0000678
Proline betaine	C_7_H_13_NO_2_	1.06295	0.009366	0.020656	HMDB0004827

To further understand the metabolic changes associated with GS, pathway enrichment analysis was performed using differential metabolites identified in serum (Figure 3(I)). The analysis highlighted glycerophospholipid metabolism (map00564), linoleic acid metabolism (map00591), and pantothenate and CoA biosynthesis (map00770) as key metabolic pathways (Table 4). The enrichment results indicated a potential disruption in lipid metabolism in the GS group.

**Table 4.** Pathway enrichment analysis results.

Pathway name	−log(p)	Impact
Glycerophospholipid metabolism	3.0762	0.21631
Sphingolipid metabolism	1.8802	0.00125
Linoleic acid metabolism	1.5485	0
Vitamin B6 metabolism	1.2976	0.07843
alpha-Linolenic acid metabolism	1.1423	0
Glycosylphosphatidylinositol (GPI)-anchor biosynthesis	1.0824	0.00639
Pantothenate and CoA biosynthesis	0.96292	0.0068
Lysine degradation	0.79777	0
Pyrimidine metabolism	0.69362	0.03985
Arachidonic acid metabolism	0.64666	0

We used ROC to assess differential metabolites for GS. Among the identified differential metabolites, two substances demonstrated strong discriminatory power with an AUC > 0.9: hexanoylglycine (AUC = 0.943) and PE (P-18:0/19:1) (AUC = 0.914). Hexanoylglycine and PE (P-18:0/19):1) were significantly downregulated in the HOM group compared to the WT group (*p <* 0.05) (Figure 3(J–M)).

## Discussion

Gitelman syndrome (GS) is an inherited renal tubular disease characterized by a complex metabolic profile involving intricate interactions among lipid, mineral, and endocrine pathways [7]. For the first time, this study uses untargeted metabolomics to reveal that, despite its classification as a renal tubulopathy, it is closely associated with dysregulated lipid metabolism. In support of a link between kidney disease and lipid metabolism, we collected serum samples from six patients diagnosed with CKD and six healthy control subjects for a non-targeted metabolomic analysis (Supplementary Figures 1 and 2, supplementary Table 2). We identified 63 differential metabolites from the non-targeted metabolism. Our findings revealed that among the 63 differential metabolites, several are implicated in lipid metabolism, including nicotinuric acid, (4E,8E,10E-d18:3)sphingosine, LysoPC(22:4/0:0), hydroxypropionylcarnitine, 2-hexenoylcarnitine, suberylglycine, and valerylcarnitine. This observation provides supplementary evidence supporting the hypothesis that renal diseases patients experience dysregulated lipid metabolism. Dysregulation of lipid metabolism is not only an important feature of chronic kidney disease (CKD) but may also play a key role in its pathogenesis [8]. For example, Impaired renal fatty acid utilization leads to lipid accumulation, triggering cellular energy deficiency and apoptosis, which, in turn, exacerbates renal fibrosis and functional decline [9].

Using WES and Sanger sequencing validation, we identified five patients with GS from four families who were homozygous for the *SLC12A3* (NM_000339.3):c.1262G > T (p.Cys421Phe) mutation. Previous studies have reported that the prevalence of growth retardation in children with GS ranges from approximately 16% to 62.5% [10,11]. Consistent with these studies, all five patients in this present study exhibited varying degrees of height retardation compared to their age-matched peers. Additionally, the mean BMI of the five patients with GS was 15.02, significantly lower than the 18.37 observed in the WT group (Figure 1(C)), suggesting that GS may negatively impact growth and development [12]. We propose that dysregulation of lipid metabolism may be a contributing factor to the developmental delays observed in patients with GS. Raw data, such as the height and weight of HOM and WT, are shown in Supplementary Table 1.

A significant association has been established between BMI reduction and decreased cholesterol levels in children and adolescents [13]. Accordingly, we prospectively evaluated serum TC levels in patients with GS. Compared to healthy subjects, patients with GS exhibited significantly lower serum TC levels (Figure 3(A)). A finding consistent with previous studies that also reported reduced cholesterol levels in patients with GS [14]. More compellingly, our *in vitro* experiments provide a cellular-level explanation for this phenomenon. To this end, we utilized a 293 T cell line harboring the *SLC12A3* (NM_000339.3): c.1262G > T (p.C421F) mutation to measure the levels of total cholesterol, free cholesterol, and cholesteryl esters in both cell lysates and supernatants (Figure 3(B–G)). The results demonstrated that the c.1262G > T mutation significantly reduced intracellular levels of total cholesterol, free cholesterol, and cholesteryl esters. Concurrently, in the cell supernatant, while total cholesterol and cholesteryl esters were also significantly decreased, the level of free cholesterol was markedly elevated. These findings suggest that the *SLC12A3* mutation leads to aberrant cellular cholesterol metabolism. We postulate that the functional defect in *SLC12A3* impairs the cell’s capacity for cholesterol esterification and storage, while promoting the active efflux of free cholesterol. This points to ‘defective cellular cholesterol trafficking’ as a key mechanism in the pathophysiology of GS, which may have significant implications for cellular function and disease pathogenesis.

We conducted untargeted metabolomics assays using LC–MS on serum samples from GS patients. The validity of the constructed model was confirmed through PCA and OPLS-DA. Applying screening criteria of VIP >1 and *p* < 0.05, we identified 27 differentially expressed metabolites. Pathway enrichment analysis of these metabolites revealed 10 GS-associated metabolic pathways, including glycerophospholipid metabolism, linoleic acid metabolism, and pantothenic acid and coenzyme A biosynthesis metabolism. Among these, glycerophospholipid metabolism (map00564) was the most significantly affected pathway, involving key differential metabolites such as PC(16:0/18:1), PE(15:1/20:4), PE(P-16:0/22:6), LysoPE(P-16:0/0:0) and LysoPC(P-16:0/0:0) (*p <* 0.05). A deeper understanding of these metabolic alterations is essential for uncovering the underlying mechanisms of kidney disease.

Lysophospholipids, such as lysophosphatidylcholine (LysoPE) and lysophosphatidylcholine (LysoPC), are intermediates of lipid metabolism generated through phospholipid hydrolysis within the cell membrane [15]. Due to their non-cylindrical structure, lysophospholipids destabilize the lipid bilayer, leading to membrane damage and intracellular K^+^ leakage, which subsequently impairs renal tubular K^+^ reabsorption [16]. L LysoPE, in particular, induces membrane depolarization and increases permeability, resulting in rapid K^+^ efflux and cell death [17]. Additionally, LysoPE may enhance membrane degradation by activating enzymes like phospholipase A_2_ [18]. Consequently, LysoPE-induced membrane damage disrupts the regulatory mechanisms of K^+^ homeostasis, ultimately affecting renal function [19].

Linoleic acid, an essential polyunsaturated fatty acid and a vital component of cell membrane phospholipids, plays a critical role in maintaining the fluidity and stability of renal cell membranes. Its deficiency may lead to renal cell damage and dysfunction, which is closely associated with the progression of CKD [20,21]. In our study, the GS group exhibited significant differences in both linoleic acid metabolism (map00591) and alpha-Linolenic acid metabolism (map00592) compared to the WT group.

Pantothenic acid, also known as vitamin B5, is a water-soluble vitamin and an essential precursor for the synthesis of coenzyme A, which plays a key role in the metabolism and synthesis of carbohydrates, proteins, and fats [22,23]. Studies have shown that pantothenic acid levels are significantly decreased in patients with diabetic nephropathy (DKD) [24]. Pantothenic acid deficiency has also been linked to a reduction in ATP synthesis in both diabetic patients and rat models [25]. Furthermore, research has demonstrated that pantothenic acid not only reduces weight gain induced by a high-fat diet in mice but also significantly improves glucose tolerance and corrects lipid metabolism abnormalities. These effects are mediated through the JNK/p38 MAPK signaling pathway, suggesting that pantothenic acid plays a protective role in maintaining lipid homeostasis and preventing metabolic disorders [26].

Metabolites, stably present in various metabolic pathways, exhibit significant correlations with disease phenotypes when the organism undergoes physiological changes. Monitoring metabolite alterations can provide insights into the effects of specific disease phenotypes on metabolic pathways [16]. We conducted an ROC analysis of differential GS serum metabolites. Based on AUC values, we identified, for the first time, that hexanoylglycine (AUC = 0.943) and PE(P-18:0/19:1) (AUC = 0.914) have potential as diagnostic biomarkers for GS. Hexanoylglycine, a fatty acid metabolite, accumulates in mitochondria, leading to the buildup of toxic intermediates that cause DNA damage and impair mitochondrial fatty acid oxidation [27]. Additionally, hexanoylglycine accumulation has been linked to obesity, with increased urinary excretion of hexanoylglycine correlating with a reduction in BMI [28]. PE(P-18:0/19:1), a type of PE, plays a critical role in lipid droplet formation, influencing both lipid storage and energy release [29]. PE enhances mitochondrial oxidative phosphorylation by modulating mitochondrial membrane fluidity and function, thereby affecting fatty acid β-oxidation [30]. For example, in a nonalcoholic fatty liver disease model, altered PE metabolism is strongly associated with reduced hepatic fatty acid oxidation, contributing to hepatic steatosis and metabolic dysregulation [31].

Recent studies have shown that IL-18 activation of the NCC protein stimulates mitochondrial fatty acid oxidation (FAO), thereby disrupting fatty acid uptake, transport, and degradation [32]. This suggests a strong association between abnormal fatty acid metabolism and GS. Furthermore, in hepatocytes exposed to high concentrations of non-esterified fatty acids and β-hydroxybutyric acid, AMPK-α expression and the transcript levels of its target genes are significantly downregulated. This suggests that PE may regulate fatty acid oxidation and synthesis through the AMPK signaling pathway, ultimately influencing mitochondrial energy metabolism [33]. Renal tubular ion reabsorption is a highly energy-demanding process [34]. Dysfunction of the NCC could lead to increased intracellular ATP consumption or decreased generation, altering the ATP/AMP ratio and subsequently activating the cellular energy sensor, AMP-activated protein kinase (AMPK) [35]. In the kidney, AMPK activity is critical for maintaining both ion transport and cellular energy balance, and its activation initiates a metabolic switch that inhibits the synthesis of fatty acids and cholesterol while simultaneously promoting the mitochondrial β-oxidation of fatty acids [36,37]. This AMPK-mediated pathway may represent an adaptive cellular response to the energy stress induced by defective ion transport in GS, ultimately leading to the systemic dyslipidemia observed in our patients. Renal tubular epithelial cells are characterized by high energy demand.

Nevertheless, we acknowledge several limitations in the present study. First, as a study focusing on a rare disease within a specific ethnic group, the sample size is relatively small, and the conclusions warrant validation in larger and more diverse population cohorts. Second, while the 293 T cell model we employed was effective for validating the functional consequences of the gene mutation, it is not a renal tubular epithelial cell line. Future studies should therefore utilize more physiologically relevant models, such as primary renal tubular cells or kidney organoids, to further investigate the underlying molecular mechanisms.

In future research, we aim to validate our findings in a larger and more demographically balanced cohort of the Yi ethnic group. We also plan to identify additional high-risk and confirmed GS patients and to include more samples with a balanced distribution of age and sex. These efforts will enable a more in-depth investigation of the specific metabolic alterations associated with the disease. In addition, we plan to conduct long-term follow-up of high-risk individuals and perform continuous metabolite monitoring to track metabolic changes preceding the onset of clinical symptoms. In a subsequent study, we will also include GS patients across different age groups to determine whether the identified differential metabolites are specific to pediatric patients or persist into adulthood. Additionally, we will track the metabolic changes in patients with GS longitudinally from childhood to adulthood. Future research will also utilize more physiologically relevant cell models, such as primary renal tubular cells, to perform in-depth investigations into the potential molecular mechanisms underlying the lipid dysregulation in patients with GS.

## Conclusions

In conclusion, this study offers novel insights into the alterations in serum metabolites associated with the pathogenesis of Gitelman syndrome (GS). Our findings reveal that the most significant differences in serum metabolites between patients with *SLC12A3* (NM_000339.3):c.1262G > T (p.Cys421Phe) mutation GS and healthy controls were related to fatty acid metabolism. This study reveals for the first time that GS is not only a renal ion transport disorder, but also a systemic lipid metabolism disorder, and the systemic manifestations of GS, such as growth retardation, provide a mechanistic explanation. We suggest that renal tubular epithelial cells, as energy-consuming cells, have NCC dysfunction that leads to cellular energy stress, which in turn activates the AMPK signaling pathway, inhibits lipid synthesis and promotes its oxidation, and ultimately triggers systemic lipid metabolism disorders. Future studies are needed to further validate the specific role of these metabolic alterations in the pathophysiology of GS.

## Supplementary Material

Supplemental Material

Supplemental Material

Supplemental Material

## Data Availability

The authors confirm that the data supporting the findings of this study are available within the article.
