# Supplementary figures and images for: Serum metabolic profiling analysis of Gitelman syndrome using untargeted metabolomics
Source: Ren Fail. 2026 Apr 29;48(1):2662094. doi: 10.1080/0886022X.2026.2662094 (PMC13130239; doi:10.1080/0886022X.2026.2662094)

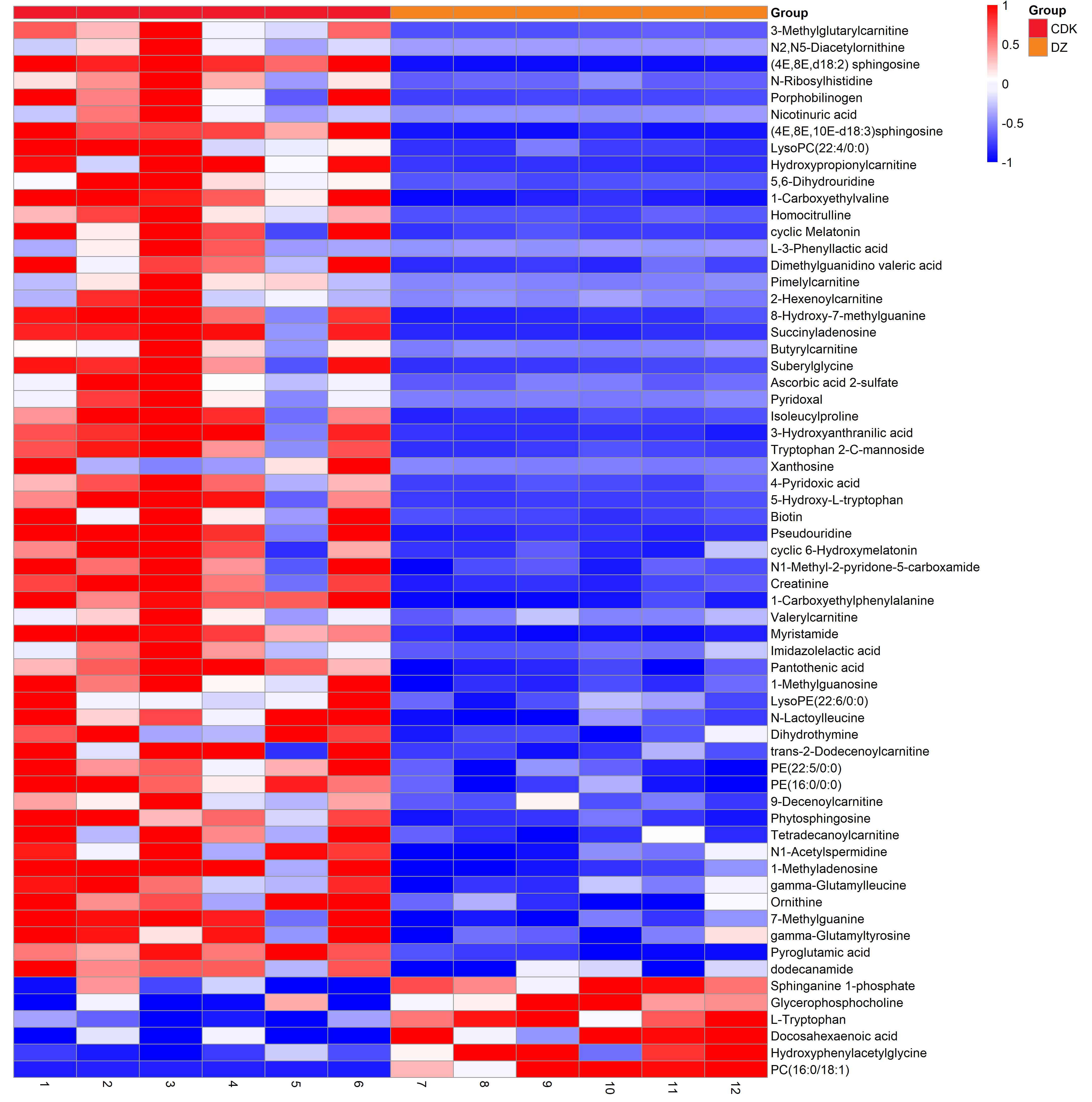

Supplement: Supplemental Material [file IRNF_A_2662094_SM7132.tiff]

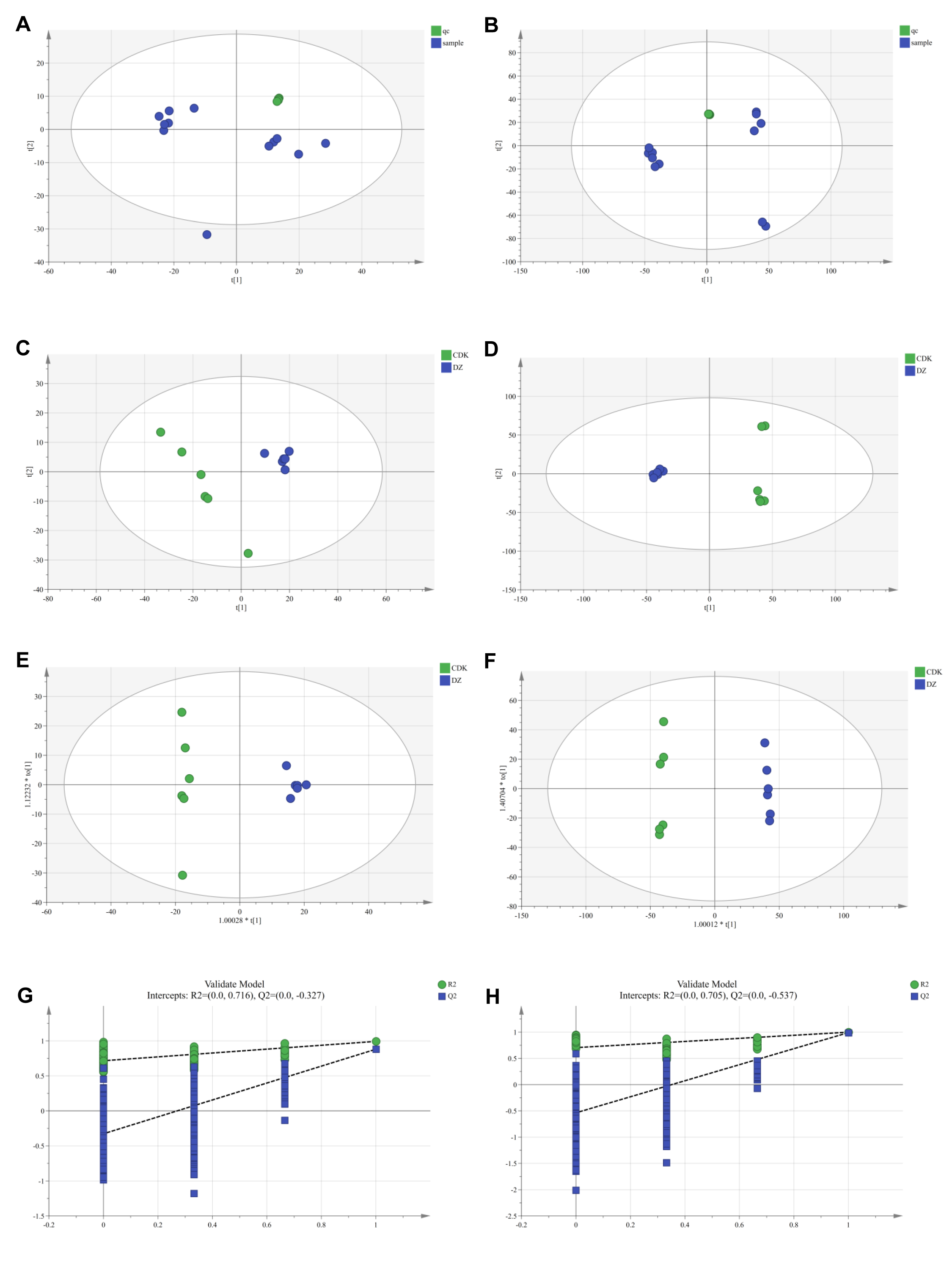

Supplement: Supplemental Material [file IRNF_A_2662094_SM7124.tif]
