# Supplementary material for: Serum metabolic profiling analysis of Gitelman syndrome using untargeted metabolomics
Source: Ren Fail. 2026 Apr 29;48(1):2662094. doi: 10.1080/0886022X.2026.2662094 (PMC13130239; doi:10.1080/0886022X.2026.2662094)
Supplement: Supplemental Material [file IRNF_A_2662094_SM7123.docx]

**Supplementary table 1** Characteristics of the study study participants

| **Individual** | **Age**  **(years)** | **Height**  **(cm)** | **Weight**  **（kg）** | **BMI**  **(kg/m^2^)** | **Total cholesterol**  **(mmol/L)** | **Gender** | **Potassium**  **(mmol/L)** | **Chloride**  **(mmol/L)** | **Magnesium**  **(mmol/L)** |
| --- | --- | --- | --- | --- | --- | --- | --- | --- | --- |
| HOM1 | 6 | 1.07 | 15.50 | 13.54 | 3.56 | Male | 2.51 | 102.30 | 0.54 |
| HOM2 | 10 | 1.30 | 26.10 | 15.44 | 4.00 | Male | 1.93 | 101.00 | 0.66 |
| HOM3 | 10 | 1.25 | 21.50 | 13.76 | 4.49 | Female | 2.11 | 99.80 | 0.52 |
| HOM4 | 13 | 1.33 | 29.00 | 16.39 | 3.77 | Male | 2.33 | 99.10 | 0.51 |
| HOM5 | 15 | 1.66 | 37.40 | 13.57 | 4.11 | Male | 2.15 | 96.30 | 0.49 |
| WT1 | 6 | 1.28 | 29.50 | 18.01 | 5.18 | Male | 4.01 | 105.00 | 0.90 |
| WT2 | 11 | 1.50 | 40.00 | 17.78 | 5.13 | Female | 4.51 | 107.20 | 1.00 |
| WT3 | 11 | 1.38 | 37.00 | 19.43 | 4.59 | Male | 4.08 | 106.20 | 0.89 |
| WT4 | 10 | 1.42 | 42.00 | 20.83 | 5.17 | Male | 4.08 | 106.20 | 0.85 |
| WT5 | 6 | 1.19 | 23.00 | 16.24 | 4.91 | Female | 3.85 | 104.20 | 0.93 |
| WT6 | 15 | 1.60 | 46.00 | 17.97 | 4.72 | Male | 4.18 | 103.70 | 0.91 |
| WT7 | 15 | 1.52 | 40.35 | 17.46 | 5.06 | Female | 4.26 | 106.50 | 0.88 |

**Supplementary table 2.** Pathway enrichment analysis results

| **Pathway Name** | **-log(p)** | **Impact** |
| --- | --- | --- |
| Vitamin B6 metabolism | 2.382 | 0.4902 |
| Glycerophospholipid metabolism | 2.1633 | 0.15981 |
| Tryptophan metabolism | 2.0047 | 0.32556 |
| Glutathione metabolism | 1.4160 | 0.00709 |
| Sphingolipid metabolism | 1.3096 | 0.00875 |
| Linoleic acid metabolism | 1.2570 | 0 |
| Biotin metabolism | 0.9675 | 0.2 |
| alpha-Linolenic acid metabolism | 0.86043 | 0 |
| Arginine biosynthesis | 0.83053 | 0.06383 |
| Nicotinate and nicotinamide metabolism | 0.80286 | 0 |
| Ether lipid metabolism | 0.68929 | 0 |
| Pantothenate and CoA biosynthesis | 0.68929 | 0.0068 |
| Porphyrin metabolism | 0.52368 | 0.0528 |
| Biosynthesis of unsaturated fatty acids | 0.46985 | 0 |
| Arginine and proline metabolism | 0.46985 | 0.16395 |
| Arachidonic acid metabolism | 0.40028 | 0 |
| Purine metabolism | 0.25426 | 0 |
